# Cardiac MR image reconstruction using cascaded hybrid dual domain deep learning framework

**DOI:** 10.1371/journal.pone.0313226

**Published:** 2025-01-10

**Authors:** Madiha Arshad, Faisal Najeeb, Rameesha Khawaja, Amna Ammar, Kashif Amjad, Hammad Omer

**Affiliations:** 1 Medical Image Processing Research Group (MIPRG), Dept. of Elect. & Comp. Engineering, COMSATS University Islamabad, Islamabad, Pakistan; 2 Dept. of Computer Engineering, National University of Technology, Islamabad, Pakistan; 3 College of Computer Engineering & Science, Prince Mohammad Bin Fahd University, Khobar, Saudi Arabia; Islamia University of Bahawalpur: The Islamia University of Bahawalpur Pakistan, PAKISTAN

## Abstract

Recovering diagnostic-quality cardiac MR images from highly under-sampled data is a current research focus, particularly in addressing cardiac and respiratory motion. Techniques such as Compressed Sensing (CS) and Parallel Imaging (pMRI) have been proposed to accelerate MRI data acquisition and improve image quality. However, these methods have limitations in high spatial-resolution applications, often resulting in blurring or residual artifacts. Recently, deep learning-based techniques have gained attention for their accuracy and efficiency in image reconstruction. Deep learning-based MR image reconstruction methods are divided into two categories: (a) single domain methods (image domain learning and *k*-space domain learning) and (b) cross/dual domain methods. Single domain methods, which typically use U-Net in either the image or *k*-space domain, fail to fully exploit the correlation between these domains. This paper introduces a dual-domain deep learning approach that incorporates multi-coil data consistency (MCDC) layers for reconstructing cardiac MR images from 1-D Variable Density (VD) random under-sampled data. The proposed hybrid dual-domain deep learning models integrate data from both the domains to improve image quality, reduce artifacts, and enhance overall robustness and accuracy of the reconstruction process. Experimental results demonstrate that the proposed methods outperform than conventional deep learning and CS techniques, as evidenced by higher Structural Similarity Index (SSIM), lower Root Mean Square Error (RMSE), and higher Peak Signal-to-Noise Ratio (PSNR).

## 1. Introduction

MRI is a non-invasive imaging modality, extensively used for the diagnosis of a variety of diseases and conditions [1–3]. However, one of its major drawbacks is the long scan time which causes patient discomfort. Also, there are physiological movements that deteriorate MR image quality in many applications of MRI e.g., Cardiac Cine Magnetic Resonance Imaging (cMRI) [4, 5].

MRI is a reference standard used to assess function of the cardiovascular system [4–8]. cMRI can be used to view the anatomical details of cardiac phases and pulmonary veins. Major challenges associated with cMRI are: (i) motion of heart, (ii) pulsatile blood flow, (iii) respiratory motion and (iv) physical motion of the patient [7]. Long scan time is also a major limitation in cMRI because assessment of cardiac structure and function requires multiple acquisitions in numerous geometrical views which increases the overall scan time of cMRI [6–8]. Moreover, the slow nature of data acquisition also makes cMRI sensitive to motion.

Under-sampling during data acquisition (acquiring partial *k*-space lines) is one way to accelerate MR imaging process which results in aliasing artifacts [3, 9]. Different image reconstruction algorithms are used to remove these artifacts. pMRI and CS-MRI provide mechanisms to reduce the aliasing artifacts which occur due to the acquisition of partial *k*-space data. CS-MRI [1, 2] is a contemporary image reconstruction method which finds an optimal reconstruction function *f*: *x* → *y* to reconstruct fully sampled MR image (y) from partial *k*-space (under-sampled) data (*x*). CS-MRI exploits image sparsity to compensate for under-sampling artifacts. Optimization problem in CS-MRI to reconstruct an image (*y*) from partial *k*-space data (*x*) can be formulated as:

$$y=\underset{y}{argmin}\|x-E\circ F(y){\|}_{2}^{2}+\lambda \|T(y){\|}_{1}$$ (1)

In the above Eq (1), *x* is the image to be reconstructed, *E* is the under-sampling operator and *F* represents the Fourier encoding operator. Similarly, *T* represents the sparsity operator, λ represents the regularization/tuning parameter and ∘ is symbol of composition. CS-MRI can be effectively integrated with pMRI which further accelerates the imaging process [4]. There are several CS-MRI and pMRI based techniques that have been proposed in the recent past e.g. Non-Linear conjugate gradient (NL-CG) and *L+S* decomposition [3, 6]. However, these techniques have limited use in high spatial-resolution applications and often lead to blurring or residual artifacts.

To overcome the limitations of conventional pMRI and CS-based methods, deep learning-based methodologies are the center of attention for many researchers these days [8, 10–14]. These techniques successfully reconstruct images from the partially acquired *k*-space data without compromising image quality [13, 14]. Recent research suggests that these algorithms have drastically outperformed the conventional Compressed Sensing (CS) based methods and yielded exceptional results.

In contrast to CS-MRI based reconstruction in Eq (1), deep learning (DL) reconstruction learns a reconstruction function *f*: *x* → *y* from large amounts of training data $\left\{\left(x^{\left(i\right)},y^{\left(i\right)}\right):i=\right.1,\cdots ,N\},$ where *f* is achieved as:

$$f=\underset{f\in U-Net}{argmin}\frac{1}{N}\sum_{i=1}^{N}{\left\|f\left(x^{\left(i\right)}\right)-y^{\left(i\right)}\right\|}^{2}$$ (2)

In Eq (2), U-Net represents deep convolutional neural network (DCNN) with some prior knowledge about image reconstruction problem determined by a training dataset that contains fully sampled MR images (termed as labels) and the corresponding under-sampled MR images (termed as inputs) [10]. U-Net preserves high-resolution features through concatenation in the up-sampling process. It also provides a low-dimensional latent representation. The reconstruction function *f* (in Eq (2)) can be viewed as the inverse mapping of the forward model *S* ∘ *F* subject to the constraints of MR images. These constraints contain prior knowledge about reconstruction problem and are assumed to exist in a low dimensional manifold. In the conventional CS-MRI framework (Eq 1), it is difficult to incorporate the complicated MR image manifold into the regularization/constraint terms. However, in the DL based reconstruction (Eq (2)), the manifold constraints learned from the training set are used to recover an artifact free reconstruction *f*(*x*) by utilizing prior knowledge on *y* [10].

It has been found in the literature that DL based MR image reconstruction can be divided into two frameworks: (i) single domain methods (image domain learning / *k*-space domain based learning) (ii) cross/ dual domain learning [14, 15]. In image domain learning method, the DL models reconstruct fully sampled MR images from the acquired under-sampled *k*-data. Similarly, in *k*-space domain-based learning, the DL frameworks learn a mapping function between the under-sampled *k-*space data and the fully sampled *k*-space data (labels) to interpolate the missing *k*-space data points [13]. Most deep learning reconstruction methods apply U-Net in image domain or in *k*-space domain [13]. Single domain DL methods discussed above utilize large training datasets to achieve the end goal of image reconstruction. However, these methods fail to exploit fully the correlation between the image and *k*-domains [13, 15].

To overcome the limitations of single domain deep learning frameworks, dual-domain frameworks have been proposed recently [14]. These frameworks exploit the correlated information between the dual domains (image and *k*-space domain) to improve the reconstruction performance. The dual-domain frameworks show superior performance compared to single domain frameworks owing to highly coupled information between the image domain and *k*-space domain.

Souza et al. proposed a dual domain DL-based MR image reconstruction framework, which consists of a *k*-space U-Net connected to an image domain U-Net [14]. However, major limitations of this work are: (i) there is no pMRI framework, (ii) the model does not have Data Consistency (DC) framework, and (iii) it was evaluated only on brain anatomy. Eo et al. proposed a dual domain DL framework termed as KIKI-net. This model cascades the *k*-space domain DL networks with the image domain DL networks interleaved by DC layers [16]. The authors of KIKI-net assess various combinations of I-net and K-net for single-domain and dual-domain reconstruction of under-sampled brain MR images. In their framework, K-net is composed of a KCNN followed by an *IFFT*, while I-net comprises an ICNN followed by an interleaved data consistency layer. A key limitation of this paper is the integration of the data consistency layer solely within the I-net, leaving K-net without it. Furthermore, the proposed method is not evaluated for its performance for pMRI reconstruction, which limits its broader applicability. Liu et al. proposed a dual domain DL framework where image domain-based DL network (termed as V-Net) and *k*-space domain-based network (termed as K-Net) are combined for Fast MRI reconstruction [13]. The authors in [13, 16] did not explore their proposed models for muti-coil (pMRI) reconstruction. Souza et al. proposed the use of a dual domain framework for the reconstruction of human brain MR images in two configurations: (a) Single Channel Configuration (SC) in which all the channels are processed independently and (b) Multi-Channel Configuration (MC) in which all the channels were simultaneously processed [14]. According to the authors in [14], hybrid approaches outperform single domain DL methods in MC configuration however, their hybrid approaches in MC configuration are not robust to different number of receiver coils. Similarly, Pramanik et al. proposed Deep Generalization of Structured Low-Rank Algorithms (Deep-SLR) [17] which effectively embodies the principles of hybrid dual-domain deep learning by merging low-rank modeling with deep neural networks, making it suitable for complex imaging tasks. However, they assessed their proposed method only on brain and knee dataset. In another work, Pramanik et al. introduced a deep monotone operator learning framework for model-based deep learning to address the inverse problems in imaging [18]. Their approach involves training a monotone convolutional neural network (CNN) within a deep equilibrium algorithm. However, their findings are currently limited to a five-layer CNN only.

In this paper, we propose a hybrid dual domain DL-based framework for robust reconstruction of highly accelerated 2D cardiac multi-coil MR data. Our paper proposes DL frameworks which employ two different combinations of hybrid dual-domain cascaded networks with interleaved multi-coil data consistency (DC) layers for the reconstruction of high-quality cardiac MR images. The reconstruction results obtained from the aforementioned proposed methods have been compared with contemporary single domain DL method utilizing U-Net [10] and CS-MRI [1, 2].

## 2. Methods and materials

In this paper, hybrid dual-domain DL frameworks for cardiac MRI are proposed by leveraging both the spatial and frequency (*k*-space) domain information. This hybrid approach can effectively capture and reconstruct fine details in MR images, leading to higher image quality. In order to avoid generalizability issues due to data acquisition from different number of receiver coils, the training of hybrid dual-domain DL frameworks has been performed on composite data (obtained from adaptive coil combination of the N receiver coil images). Moreover, the multi-coil data consistency layer (MCDC) has also been integrated in our proposed hybrid dual-domain DL frameworks. The MCDC layer provides data consistency across multiple coils and ensures an accurate spatial and frequency domain representation, reducing the likelihood of reconstruction errors and leading to more reliable and accurate final images.

**Fig 1:**
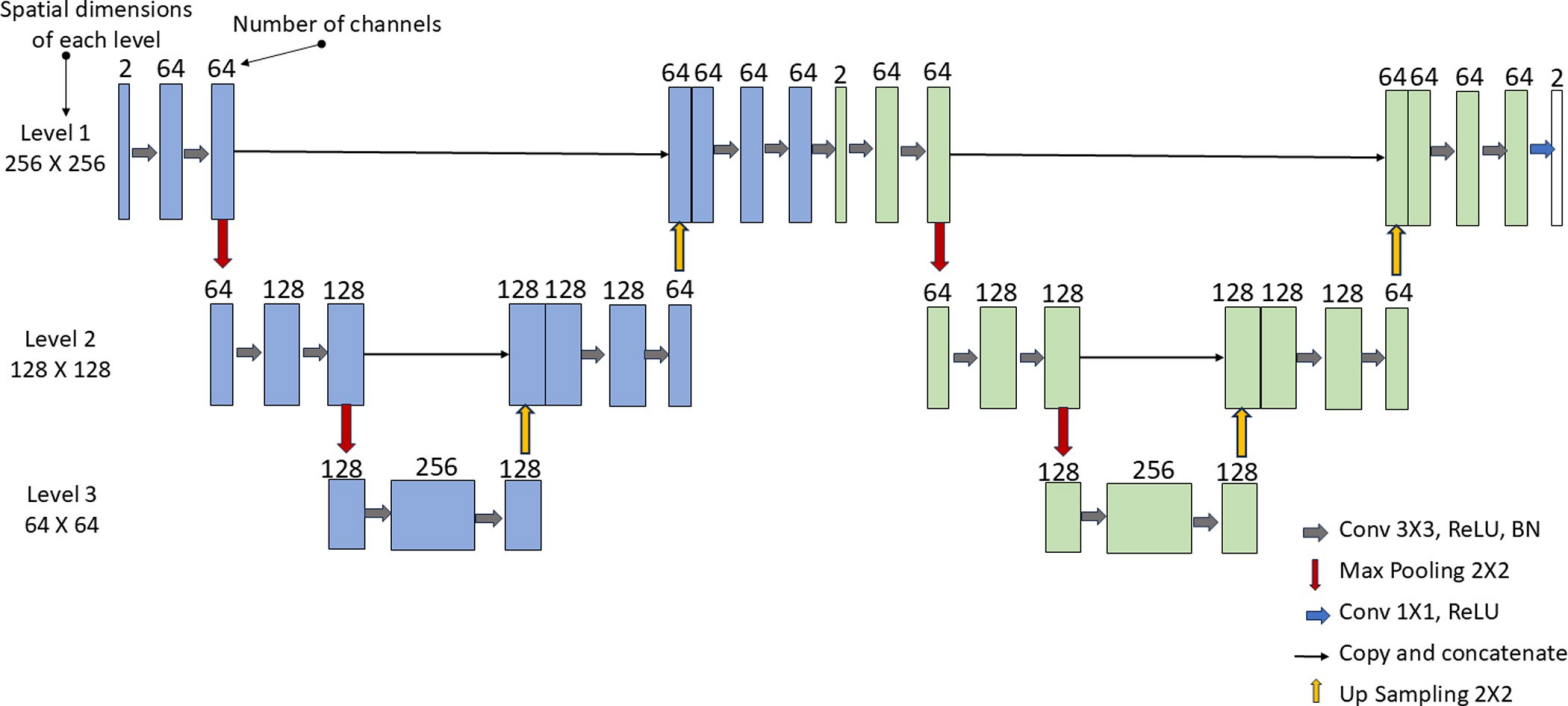
An architecture of customized cascaded U-Net used in the proposed methods 1 and 2.

The proposed DL frameworks consist of an architecture which has been created by cascading two customized U-Nets [10]. Fig 1 provides an overview of the architecture of cascaded U-Nets used in our proposed frameworks. The customized architecture of U-Net in our proposed method contains 10 convolution layers (followed by Rectified Linear Unit (ReLU) and Batch Normalization (BN) having filter size of 3×3 and the last convolution layer (followed by BN) having filter size of 1×1. It also contains max pooling and up sampling layers. The proposed frameworks consist of two cascaded U-Nets; therefore, there are two times as many convolutional, max pooling, up sampling layers and other trainable parameters.

The proposed methods 1 and 2 investigate the possible dual domain configurations of our proposed frameworks i.e., KI-Net and IK-Net respectively, in order to reconstruct the complex-valued zero-filled variable density (VD) under-sampled human cardiac MRI data at different Acceleration Factors (AFs). In proposed methods 1 and 2, the training of KI-Net and IK-Net was performed in an incremental manner.

In this paper, training dataset is extracted from the fully sampled, multi-slice, eight receiver coils (Cartesian) human cardiac data of fifteen patients [19]. The fully sampled human cardiac *k*-space data is VD under-sampled by an acceleration factor (AF) of 2 and 4 retrospectively; followed by an adaptive coil combination to get the composite under-sampled *k*-space data. In our proposed methods, zero filled VD under-sampling pattern with Gaussian smoothing [4] is used to under-sample the fully sampled *k*-data at different AFs.

### 2.1. Proposed method 1 (KI-Net)

**Fig 2:**
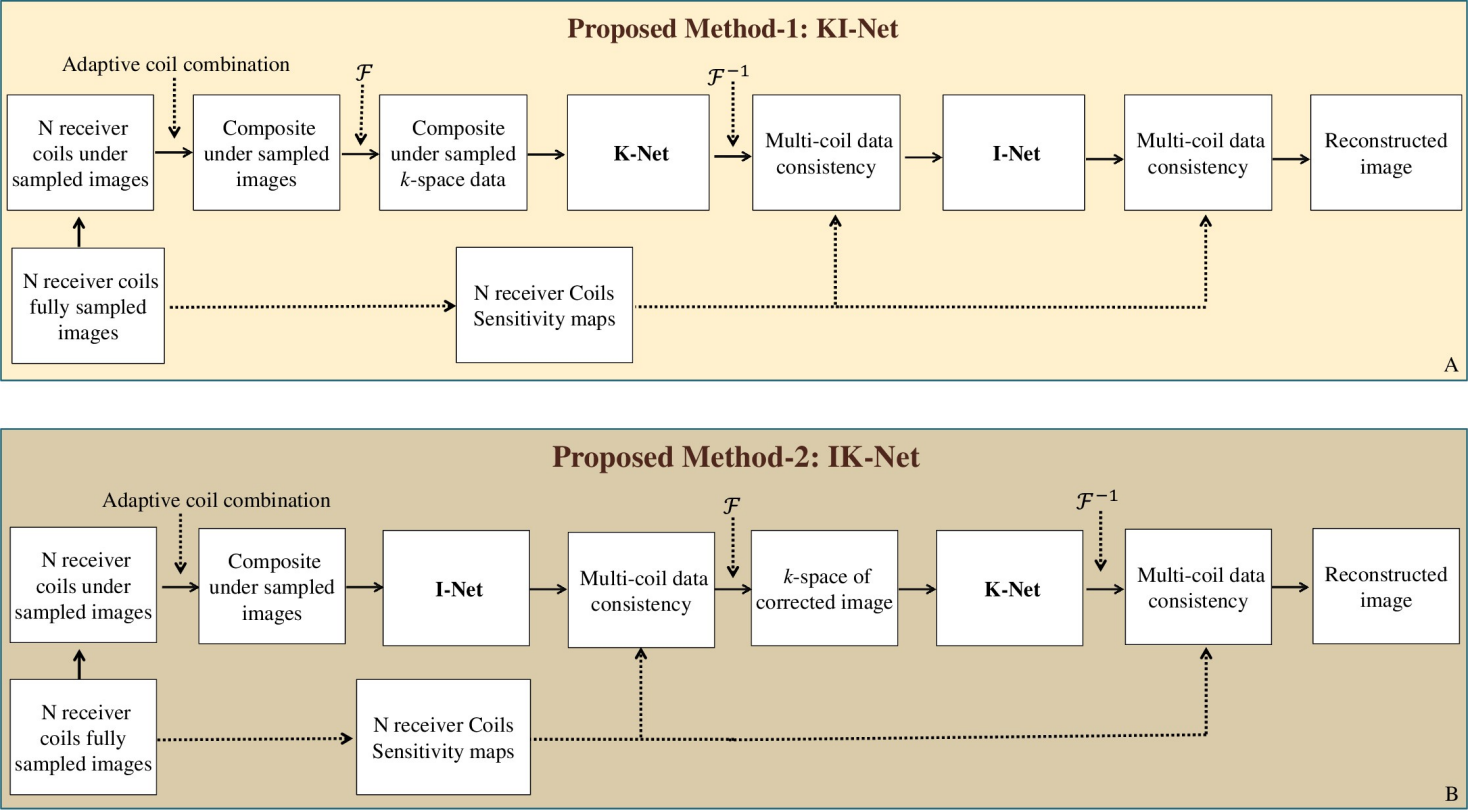
Block diagrams of Proposed Methods 1 and 2: ‘A’ presents the block diagram of Proposed Method 1 i.e. KI-Net. In this method, composite images obtained through adaptive coil combination from N receiver coils’ VD under-sampled images are fed into K-Net. K-Net generates interpolated composite k-space data, which undergoes inverse Fourier transform to produce the interpolated image. This image, after being processed by MCDC, is then given to I-Net. The final reconstructed image is produced by I-Net, followed by another MCDC operation. ‘B’ presents the block diagram of Proposed Method 2 i.e. IK-Net: In this method, the composite images are first fed to I-Net. I-Net generates artifact-free solution images, which are then processed by the MCDC layer. The output from the MCDC operation is the corrected image, which is subsequently given to K-Net after applying FFT. The final reconstructed image is produced by K-Net, followed by another MCDC operation.

The proposed method 1 (KI-Net) consists of an architecture which has been created by cascading two customized U-Nets (termed as KI-Net in this paper) as shown in Fig 2A. The proposed method 1 (KI-Net) consists of two subnetworks; named as ‘K-Net’ and ‘I-Net’. The first subnetwork (i.e., K-Net) is trained in *k* domain followed by Multi-coil Data Consistency (MCDC) operation; and then the output is fed into the second subnetwork (i.e., I-Net) after applying inverse Fourier transform followed by the second layer of MCDC operation.

For the Proposed Method 1, fully sampled, multi-slice, 8 receiver coils human cardiac data of fifteen patients is used as training dataset [19]. This fully sampled multi-slice 8 coil human cardiac *k*-data is under-sampled by an AF of 2 and 4, respectively; followed by an adaptive coil combination [20] to get the ‘coil combined’ under-sampled *k*-space data.

This under-sampled (coil combined) *k*-space data is used as an input whereas the corresponding fully sampled (coil combined) *k*-space data is used as the label for training the K*-*Net (subnetwork-1 in proposed method 1). For training of the K-Net, the real and imaginary parts of the complex *k*-space data are concatenated along the channel dimension.

**Fig 3:**
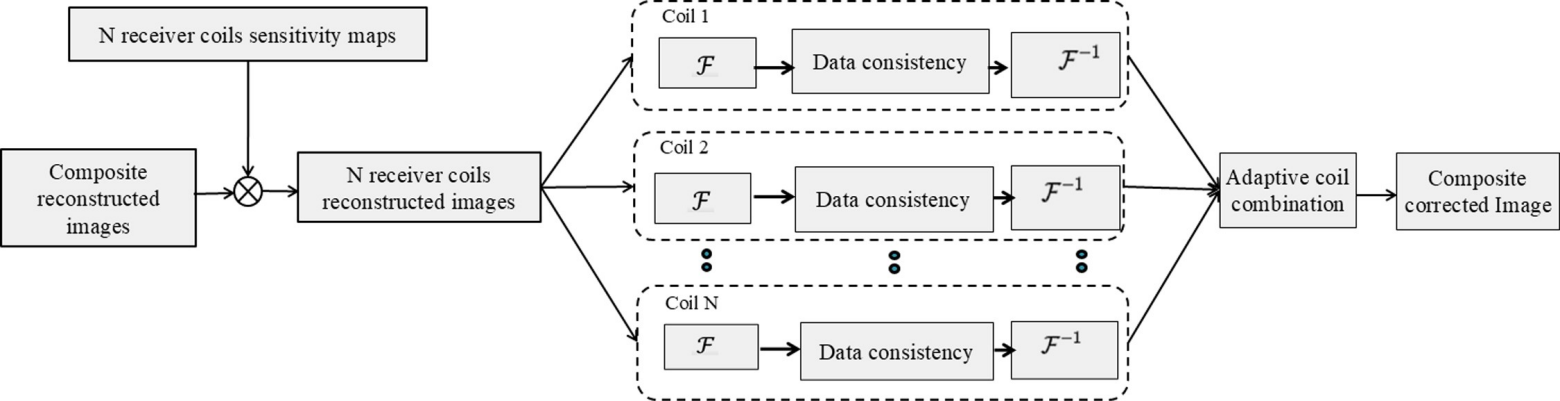
Multi-coil data consistency (MCDC) operation: First the composite reconstructed images(output of neural network) are multiplied with the N-receiver coil sensitivity maps (obtained by Walsh method [20]) in order to generate N-receiver coils reconstructed images. Further, data consistency operation is applied on each individual coil to generate the N receiver coils corrected images. Adaptive coil combination [20] of N receiver coils corrected images generates the coil combined corrected images.

The output of this trained subnetwork-1 in the proposed method 1 (KI-Net) is an interpolated complex *k*-space data. Application of inverse fast Fourier transform (*IFFT*) on the interpolated complex *k*-space data provides interpolated MR images. MCDC operation (shown in Fig 3) is applied on the interpolated images to generate the corrected images. In MCDC operation, receiver coils sensitivity maps (estimated by Walsh method [20]) are used to generate the *N* receiver coils interpolated images. Fourier transform is applied on each coil image followed by MCDC operation [14]. This provides the corrected *k*-space data of *N* receiver coils. The inverse Fourier transform of *N* receiver coils corrected *k*-space data; followed by an adaptive coil combination [20] results in the coil combined corrected images as shown in Fig 3.

These combined corrected images are given as an input to the I-Net (subnetwork-2 in proposed method 1) as shown in Fig 2A which removes the remaining aliasing artifacts. The I-Net is trained by using the corrected images as an input and the corresponding fully sampled images as the ground truth labels. For the training of I-Net, the real and imaginary parts of the input and label images are concatenated along the channel dimension. The MCDC operation is applied again on the output of I-Net to get the final reconstructed image from the proposed method 1 (KI-Net).

In Proposed Method 1, the training of the both subnetworks i.e., K-Net and I-Net is performed separately. During training of K-Net and I-Net, the convolution layers weights are initialized by a normal distribution. There was no bias term in the normal distributed weights. These weights are later optimized by minimizing the loss (Mean Square Error) function between the estimated output obtained from the sub networks (i.e., K-Net and I-Net) and the corresponding labels of the neural sub-networks (K-Net and I-Net). This *l*_2_ loss function is minimized by using RMSProp optimizer. The learning rate for this optimizer was set to 0.001 initially. This learning rate of RMSProp optimizer is further reduced by a factor of 0.1 for the patience of 10 epochs. In our experiments, the neural networks require total training time of 16 hours (using an early stopping criteria) on Python 3.7.1 by Keras using Tensor Flow as a backend. All the experiments are performed on Intel(R) Core (TM) i7-4790 CPU having 16 GB RAM and NVIDIA GeForce GTX 780 GPU. The Proposed Methods 1,2 are tested on 500 human cardiac images [19] of five patients obtained from a 1.5T scanner; as well as on the clinically acquired multi-coil human cardiac OCMR dataset [21].

### 2.2. Proposed method 2: (IK-Net)

The Proposed Method 2 (Fig 2B) uses two subnetworks i.e. I-Net (followed by MCDC operation) and K-Net (followed by MCDC operation) to reconstruct the complex-valued zero filled variable density (VD) under-sampled human cardiac data (AF = 2 and 4).

Block diagram of the proposed method 2 (IK-Net) is shown in Fig 2B. For the proposed method 2, training dataset is extracted from the fully sampled, multi-slice, eight receiver coils (Cartesian) human cardiac data of fifteen patients [19]. The fully sampled human cardiac *k*-space data is VD under-sampled by an acceleration factor (AF) of 2 and 4 retrospectively; followed by an adaptive coil combination to get the composite under-sampled *k*-space data. *IFFT* of the composite under-sampled *k*-space data provides the aliased human cardiac images which are given as an input whereas the corresponding coil combined fully sampled human cardiac images are used as the ground truth for training the I-Net (sub network-1 of the proposed method 2).

The output of the trained subnetwork-1 (I-Net) is the reconstructed image which is given as an input to the MCDC operation. In MCDC operation, sensitivity maps obtained by the Walsh method [20] are used to apply data consistency on the multi-coil interpolated images. These are later ‘coil combined’ to get the corrected composite images and then the fast Fourier transform is applied to achieve the corrected *k*-space data. The MCDC operation has been described in detail in Fig 3.

The K-Net (subnetwork-2 of proposed method 2) is trained by using the corrected *k*-space data (output of I-Net followed by MCDC operation and *FFT*) as an input and the corresponding fully sampled *k*-space data as the ground truth label. The inverse Fast Fourier transform (*IFFT*) of the output of K-Net provides solution images. MCDC operation (shown in Fig 3) is applied on the solution images to generate the final reconstructed image.

The parameters for training the neural networks had the same attributes as mentioned in Section 2.1. The Proposed Method 2 is tested on 500 human cardiac images of five patients obtained from a 1.5T scanner [19] as well as on the clinically acquired multi-coil human cardiac dataset of OCMR [21].

## 3. Datasets

The neural networks in the proposed methods 1 and 2 are trained to recover the artifact-free images from the aliased images. In the training dataset, 80% of the total images were used for training purposes and 20% were used for validation purposes. For training, each 4D cardiac dataset [N_x_, N_y_, slice, cardiac phase] was converted to 3D training examples [N_x_, N_y_, slice*cardiac phase] where N_x_, N_y_ show the number of rows and columns, respectively. The proposed methods 1 and 2 were later tested on the clinically acquired multi-coil human cardiac dataset (OCMR) [21].

### 3.1 Simulated dataset: 8 receiver coils human cardiac dataset

The dataset on which the model is trained and fitted comprises short-axis human cMRI data downloaded from https://jtl.lassonde.yorku.ca/software/datasets/. The data from 15 patients was used for training the model. The data was provided by the Diagnostic Imaging Department of Sick Children’s Hospital located in Toronto, Canada [19]. Each image slice of this cardiac dataset contains 256 × 256 pixels with a pixel-spacing of 0.93–1.64 mm.

This was single coil dataset which was simulated into 8 receiver coils by utilizing sensitivity maps information of 8 receiver coils obtained from Biot-Savart law [20]. This simulated 8 receiver coils human cardiac data was VD under-sampled by an AF of 2 and 4, respectively. The proposed methods 1 and 2 were later tested on five subjects, comprising a total of 500 2D cardiac images.

### 3.2 Clinical dataset: 18 receiver coils human cardiac data (Table 1)

**Table 1:** Details of cardiac datasets used in our experiments for proposed methods 1 and 2. For the training and testing purpose, we used 8 receiver coils human cardiac (simulated) dataset. Similarly, for testing the proposed methods, we also used multi-coil human cardiac clinical dataset, publicly available from Ohio State University [21].

Sr. no	Dataset	No. of Receiver Coils	No. of Cardiac Cycles	Magnetic field strength of the scanner	Pulse Sequence	No. of Slice
1	Simulated dataset [19]	8	20	1.5 T GE Genesis Signa scanner	FIESTA scan protocol	15
2	Clinical data: OCMR dataset [21]	18	18	3 T Siemens MAGNETOM scanner, Prisma	Asymmetric spin echo	1

OCMR is a publicly available multi-coil dataset for cMRI [21], available at: https://github.com/MRIOSU/OCMR. For testing the proposed methods 1 and 2, we used this OCMR cardiac dataset which comprises of 18 receiver coils and 18 frames, respectively. The images were cropped keeping in view the selected Region of Interest (ROI) of the tissue boundary between the left and right ventricles in the myocardium. The images were then interpolated using bi-cubic interpolation to restore the original dimensions of the images. This dataset is used to generate 18 images for the testing purpose.

### 3.3 Performance evaluation

SSIM, RMSE and PSNR are used to evaluate the reconstruction results of the proposed methods [22, 23]. Adaptive coil combination [20] of the multiple receiver coils fully sampled human cardiac images were used as the reference image.

## 4. Results

**Fig 4:**
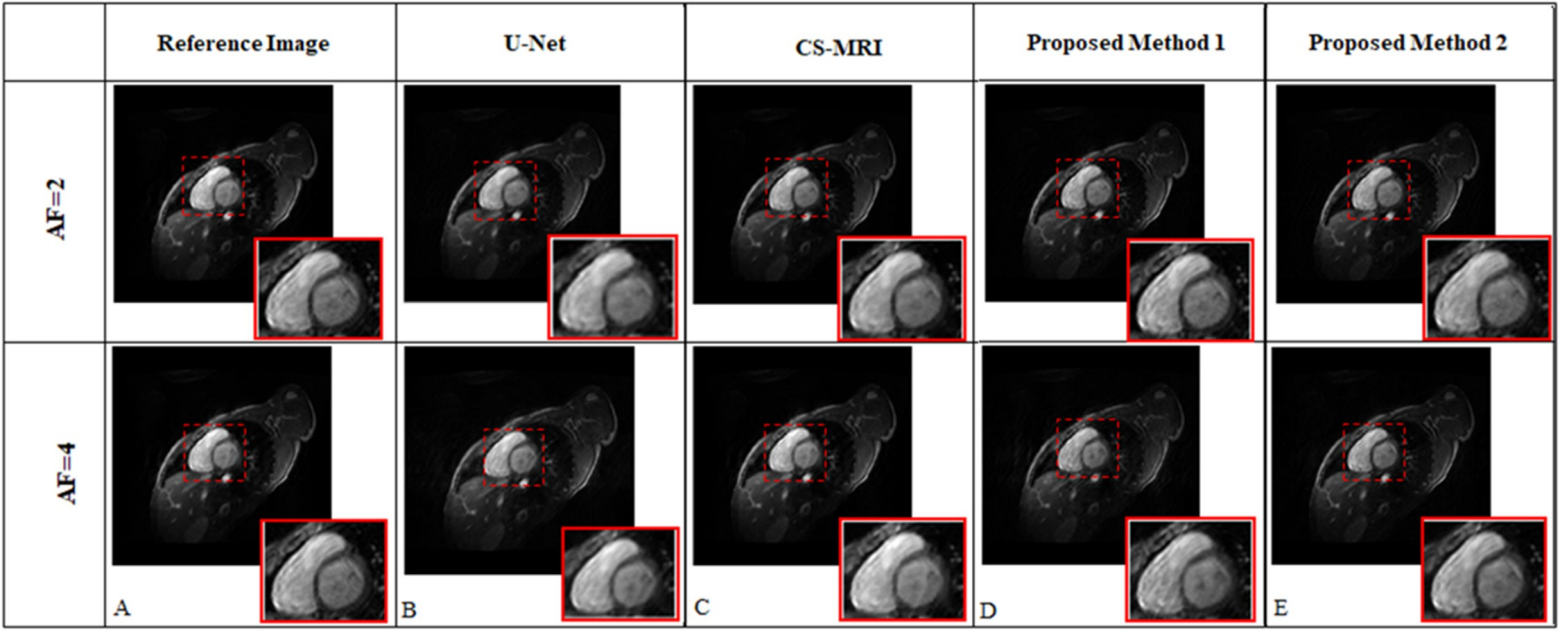
Reconstructed human cardiac images obtained from proposed methods 1 and 2 (KI-Net and IK-Net), U-Net and CS-MRI. Column ‘A’ presents the reference image which is obtained by taking the adaptive coil combination [19] of the fully sampled multi-coil human cardiac data. Columns ‘B-E’ show reconstructed images of conventional U-Net, CS-MRI, Proposed Method 1 and Proposed Method 2, respectively. It is clear from Fig 4 that Proposed Method 2 (IK-Net) shows better reconstruction results (reconstructing myocardium) of the simulated cardiac dataset than Proposed Method 1, U-Net and CS-MRI.

Fig 4 shows the reconstruction results obtained from the proposed methods (KI-Net and IK-Net) compared to conventional U-Net [10] and CS-MRI [1, 2, 24, 25] reconstruction for human cardiac dataset-1 at different AFs. These are the reconstruction results when the proposed methods were tested on simulated dataset i.e., 8 receiver coils human cardiac dataset-1 [19]. The column A in Fig 4 shows the label (reference) image. The columns ‘B-E’ show the reconstruction results obtained from U-Net, CS-MRI, proposed methods 1 and 2 (KI-Net and IK-Net), respectively.

As highlighted in Fig 4, the anatomical region (myocardium) is reconstructed better in the case of Proposed Method 2 as compared to Proposed Method 1 and resorted to less blurring comparatively. For the case of AF = 4, the anatomical region is sharper as compared to that of Proposed Method 1 (KI-Net), U-Net and CS-MRI. From visual assessment, we conclude that cascading the network in the order such that neural network trained in image domain (I-Net) followed by MCDC layer and then *k*-space domain (K-Net) yields better results. This is because I-Net and the global property of Fourier transform yields the corresponding *k*-space data which has now recovered the missing data points; allowing K-Net to perform better [14, 15]. The final MCDC layer allocates the originally acquired data points and retrospectively places them at their corresponding reference points, which further improved our results.

Table 2 shows the quantifying parameters (i.e., RMSE, SSIM and PSNR values) of the reconstruction results of the proposed methods 1, 2, U-Net and CS-MRI. Table 2 shows that the RMSE, PSNR and SSIM values of the proposed method 2 (IK-Net) are 0.007, 42.37 (db) and 0.9713 for simulated human cardiac data (dataset-1) at AF = 2. This quantitative analysis in Table 2 supports our visual assessment i.e. Proposed Method 2 (IK-Net) performs better as compared to Proposed Method 1 (KI-Net), U-Net and CS-MRI.

**Table 2:** Comparison of quantifying parameters (i.e., SSIM, PSNR and RMSE values) of the proposed methods 1, 2, U-Net and CS-MRI for dataset-1(simulated data) and dataset-2 (clinical data).

Datasets	AF	Method	SSIM	PSNR (dB)	RMSE
**Simulated Dataset**	2	Proposed Method 1	0.965	40.82	0.0091
		Proposed Method 2	0.974	42.37	0.007
		U-Net	0.94	37.41	0.0107
		CS-MRI	0.900	33.11	0.0100
	4	Proposed Method 1	0.907	34.83	0.018
		Proposed Method 2	0.94	38.77	0.0115
		U-Net	0.90	33.19	0.0219
		CS-MRI	0.85	29.81	0.0220
**OCMR Dataset**	2	Proposed Method 1	0.95	40.31	0.0097
		Proposed Method 2	0.94	37.31	0.012
		CS-MRI	0.89	35.53	0.014

**Fig 5:**
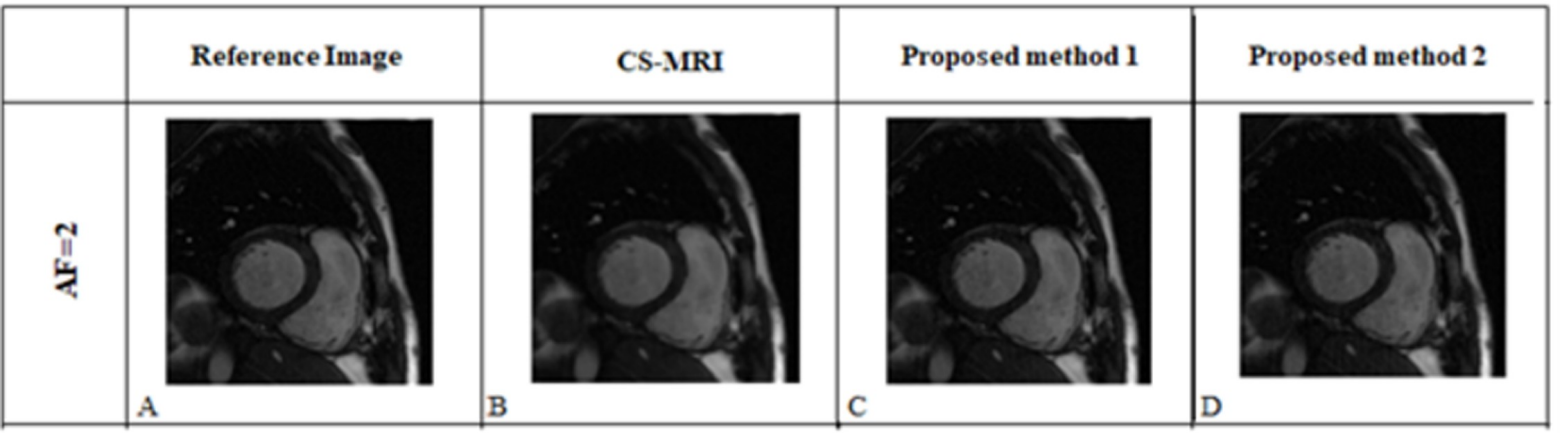
Reconstructed cardiac images of CS-MRI, proposed methods 1 and 2 for clinical dataset (i.e., 18 receiver coils human cardiac data). Column ‘A’ presents the reference image which is obtained by taking the adaptive coil combination [20] of the fully sampled 18 receiver coils human cardiac data. Columns ‘B-D’ show the reconstructed images of CS-MRI, Proposed Methods 1and 2, respectively.

The proposed methods were also tested on clinically acquired datasets as mentioned in Section 3.2. Fig 5 demonstrates the reconstruction results of the clinical dataset i.e. 18 receiver coils human cardiac data set (OCMR dataset) [21]. Table 2 also provides SSIM, RMSE and PSNR values of the proposed methods and CS-MRI for OCMR dataset. We further reiterate the presented analysis that the order of training yields better output results if placed in *I*-*K* order i.e., proposed method 2 (IK-Net) compared to CS-MRI and proposed method 1 (KI-Net).

Table 3 shows SSIM, PSNR and RMSE values of the reconstructed images of Proposed Methods 1 and 2. These quantitative assessment values are reported as mean ± standard deviation (STD) across the test images of a specific testing dataset. For quantitative assessment, the proposed methods have been tested on 500 images of simulated data [19] and 18 images of clinical OCMR dataset [21].

**Table 3:** Comparison of SSIM, PSNR and RMSE values of the reconstructed images from the proposed methods 1 and 2 (KI-Net and IK-Net). We reported these values as mean ± STD across the test images of a simulated and OCMR datasets.

AF	Method	SSIM	PSNR (dB)	RMSE
**Testing Dataset 1 = Simulated Data (Test Images = 500)**				
**2**	Proposed Method 1	0.95±0.02	41.73±2.96	0.0086±0.002
	Proposed Method 2	0.95±0.013	43.37±0.017	0.0072±0.001
**4**	Proposed Method 1	0.85±0.044	34.95±2.26	0.0196±0.005
	Proposed Method 2	0.90±0.026	38.32±0.026	0.0122±0.002
**Testing dataset 2 = OCMR data (Test Images = 18)**				
**2**	Proposed Method 1	0.93±0.002	38.20±0.29	0.0123±0.0004
	Proposed Method 2	0.957±0.004	40.00±0.75	0.0106±0.008

## 4. Discussion

CS-MRI [1, 2, 24] is widely used to reconstruct fully sampled MR images from the acquired under-sampled MRI *k*-data. CS-MRI has recently been introduced on clinical MRI scanners [25]; however, there are some limitations associated with CS-MRI. Deep learning-based MR image reconstruction is the center of attention for many researchers these days. Most deep learning reconstruction algorithms use U-Net or its variants [8, 10]. Furthermore, most deep learning reconstruction methods apply U-Net in the image domain or in k-space domain. Single domain DL methods discussed above utilize large training datasets to achieve the end goal of image reconstruction. However, these methods fail to exploit fully the correlation between the image and *k*-domains [13, 15].

This research work evaluates the effectiveness of hybrid dual domain deep learning frameworks in reconstructing the multiple receiver’s coils cardiac MR images. For a fair comparison, we compared our proposed methods with CS-MRI and U-Net. Compared to conventional U-Net [10] and CS-MRI, the proposed methods provide better reconstruction results.

The existing deep learning methods outperform contemporary CS-MRI methods because of two properties: (i) data-driven feature extraction and (ii) high nonlinearity [26–30]. However, the major limitation of majority of the deep learning frameworks discussed in the previous literature is that they are only trained in the image domain. These frameworks reconstruct artifact free images from data in which the detailed structures are already distorted or have even disappeared [27–29].

Moreover, single domain neural networks are less effective in simultaneously extracting features in dual domains (image domain and *k*-space domain) [13]. Hybrid dual domain deep learning framework is effective in resolving the aforementioned problem because the deep learning-based network that trained in the *k*-space can exploit *k*-space more efficiently to recover the missing data. In this research, we iteratively applied two different deep learning networks operating in different domains (one in *k*-space and the other in image domain). MCDC operation was interleaved between the deep learning networks.

The proposed methods consist of the following three components: First component is K-Net for *k*-space interpolation, second component is I-Net for removing artifacts and restoring image details, and the third component is MCDC operation for regularizing the network training. Moreover, for iterative optimization, each deep learning network is alternatively applied and independently trained. This provided two configurations for hybrid dual domain: KI-Net and IK-Net. Although I-Net and K-Net have been trained on ‘coil combined’ images, our proposed deep learning frameworks incorporate receiver coil sensitivity maps which provide multi-coil reconstruction.

In our experiments, I-Net and K-Net have been trained on simulated multi-coil data. This is because deep learning algorithms need a larger cohort of data to train the deep learning framework and to achieve robust generalization performances [31]. However, it is difficult to get large number of multi-channel data for model training since saving the raw *k*-space data is not included in the routine clinical procedures [32]. In order to tackle this data collection problem, the authors in [33] suggested using simulated images in the training dataset. We used simulated multi-coil data for the training and testing purposes. For generating the simulated multi-coil data, sensitivity maps estimated by Biot Savart law [20] have been used.

Fig 4 shows the reconstruction results of the proposed methods, U-Net [10] and CS-MRI [1, 2] for simulated data (dataset-1); with a focus on the reconstruction of myocardium (the tissue boundary between the left and right ventricles of the heart). Fig 5 shows that the anatomical details of the reconstructed images of Proposed Method 2 are more precise as compared to Proposed Method 1. In all our experiments (as shown in Figs 4 and 5 and Tables 2 and 3), IK-Net (Proposed Method 2) model outperformed KI-Net (Proposed Method 1) model indicating that it may be advantageous to use an image domain network first in cascaded dual domain networks. Similar results were also reported in [14, 15]. This is because high frequency components of partial *k*-space data (under sampled data) are less densely sampled, which results in regions where the convolutional kernel would have no signal to operate upon. By starting with an image domain block in Proposed Method 2 (i.e., IK-Net), because of the modulation property of Fourier Transform (FT), the output of this network has a corresponding *k*-space that is now evenly distributed, effectively catering the problem of regions with no samples for the convolution kernels to operate on [14, 15].

We also evaluated the proposed methods on clinically acquired human cardiac multi-coil dataset as shown in Fig 5. This human cardiac data has been acquired using 18 receiver coils. Our proposed deep learning frameworks (Proposed Methods 1 and 2) which were initially trained on the reconstruction of 8 receiver coils human cardiac data successfully reconstructed the 18 receiver coils human cardiac data (as reflected by their RMSE, PSNR and SSIM values shown in Table 2). The reconstruction results shown in Figs 4 and 5 and Tables 2 and 3 indicate the robustness of our proposed methods in reconstructing the human cardiac data acquired using different number of receiver coils. This is because our proposed frameworks do not learn MCDC during training. Moreover, training of the neural networks was also performed on ‘coil combined’ images. This helps our proposed deep learning algorithms in avoiding the generalization issues that arise when we use training and testing data acquired using different number of receiver coils.

Overall, the proposed dual-domain learning allows the neural network to learn more robust features by simultaneously considering the spatial and frequency domain data, improving its overall performance in image reconstruction. The proposed dual-domain approach improves the model’s ability to generalize data acquired using different number of receiver coils, making it more reliable in clinical practice. The hybrid dual-domain frameworks may serve as a foundation for integrating more advanced deep learning techniques, such as reinforcement learning [34] or transfer learning [31, 32] to further improve MRI reconstruction. In future, the attention mechanism [35, 36] and motion correction framework [36–38] could also be integrated in the proposed methods.

## 5. Conclusion

The proposed hybrid dual domain deep learning frameworks successfully reconstruct the cardiac MR images from the acquired highly under-sampled *k*-space data. Visual assessment of the images reconstructed images confirms that the proposed methods provide images comparable to the fully sampled reference images. The proposed frameworks are more flexible as compared to single domain U-Net [10] as they are independent of the number of receiver coils.

The results show superior reconstruction from the proposed methods than conventional methods as reflected by SSIM, RMSE and PSNR values e.g., there is a 3.49% and 7.59% improvement in SSIM values of the Proposed Method 2 compared to U-Net [10] and CS-MRI [1, 2] reconstructions respectively for simulated cardiac data (dataset1) at AF = 2. Similarly, there is 12.41% and 21.85% improvement in PSNR values of the Proposed Method 2 compared to the conventional U-Net and CS-MRI reconstructions respectively for cardiac data at AF = 2 in our experiments.
